# A Review on Basalt Fiber Composites and Their Applications in Clean Energy Sector and Power Grids

**DOI:** 10.3390/polym14122376

**Published:** 2022-06-12

**Authors:** Hechen Liu, Yunfei Yu, Yunpeng Liu, Mingjia Zhang, Le Li, Long Ma, Yu Sun, Wanxian Wang

**Affiliations:** 1 Hebei Key Laboratory of Distributed Energy Storage and Micro-Grid, North China Electric Power University, Yonghua North Street No. 619, Baoding 071003, China; yyf990203@163.com (Y.Y.); liuyunpeng@ncepu.edu.cn (Y.L.); 18367683603@163.com (M.Z.); lile@ncepu.edu.cn (L.L.); sunyu1777885536@163.com (Y.S.); 220192213050@ncepu.edu.cn (W.W.); 2 State Key Laboratory of Alternate Electrical Power System with Renewable Energy Sources, North China Electric Power University, Beinong Road No. 2, Beijing 102206, China; mayida1988@163.com; 3 Key Laboratory of Physical and Chemical Analysis for Electric Power of Hainan Province, Hairui Road No. 23, Haikou 570100, China

**Keywords:** basalt fiber, composite material, surface modification, electrical materials

## Abstract

Basalt fiber (BF) has a high mechanical strength, excellent temperature resistance, good chemical stability, low energy consumption, and an environmentally friendly production process. In addition, BF-reinforced polymers (BFRPs) have good corrosion resistance and designability; thus, they meet the application requirements of electrical equipment, such as new conductors, insulating pull rods, and composite cross-arms. However, there are still a series of technical issues in the mass production of BF, and the stability of the products needs to be further improved. Therefore, the research on the production, modification, and application of BF is necessary. This paper discusses the chemical composition and production technology of BF, describes the morphology and properties of BF, summarizes the interface problems and modification methods of composites, and finally, introduces the application prospects of BF in the field of electrical materials, which is expected to provide a reference for the application and promotion of BFRP in the future.

## 1. Introduction

Basalt fiber (BF) is a high-performance inorganic silicate fiber made from natural basalt ore by high-temperature melting and drawing, and it is a kind of green fiber that does not create environmental pollution or pose a cancer risk [1,2,3]. Compared with traditional glass fiber (GF), BF has better mechanical properties, as well as high-temperature and corrosion resistance [4]. Compared with carbon fiber (CF), BF has a lower cost and is one of the best choices to replace GF and CF [5,6]. Therefore, basalt fiber-reinforced polymers (BFRPs) have been widely used in the petrochemical, construction, aerospace, automobile, ship, and other industries [7,8,9].

In 1922, a Frenchman named Paul Dhé proposed the manufacturing technology of BF, but no actual production was carried out [10]. In 1954, BF was successfully extracted from basalt ore by the Soviet Union [11]. In 1985, the Soviet Union realized the industrial production of BF for the first time in Ukraine and applied the products to the national defense industry. However, the production process was poor, the equipment structure was complex, and the energy consumption was high at that time. In the late 1990s, the Soviet Union successfully developed a new-generation BF production process and equipment, which reduced the energy consumption by 2.5 times [12,13]. In China, the research on BF has been carried out since the 1970s. It was not until the 1990s that progress was made, and self-made BF was used to prepare thermal insulation materials and fighter shells. At the beginning of the 21st century, China mastered and perfected the production technology of BF, and various categories of products were developed, and BF was listed in the National 863 plan. Currently, the main manufacturers involved in the production and marketing of BF worldwide include Kamenny VeK of Russia, OJSC Institute of Glass Plastics and Fibers in Ukraine, and Shanghai Ruskin Basalt Fiber Company of Hengdian Group in China. Of these, Hengdian Group has performed a considerable amount of work on the preferential selection of basalt ore, design of basalt melting furnaces, selection of the heating process, and introduction and development of the drawing process since its establishment, and it has been ahead of other enterprises in China on the research of BF.

In the power industry, electrical equipment such as new conductors, insulating pull rods, and composite cross-arms are mostly made from glass-fiber-reinforced composite materials. However, with the continuous development of the power grid, voltage levels and the installed capacities of generators continue to increase. The traditional GF has some disadvantages, such as having a low elastic modulus, being brittle and easy to crack, and readily aging in hot and humid environments, and it has gradually become unable to meet the application requirements of some electrical equipment in complex environments. For example, in humid and hot coastal areas, the corrosion of GF composite cross-arms and composite poles is severe, resulting in a significant decline in their tensile strengths and service lives. When the length of wind turbine blade is more than 40 m, GF composite materials have been unable to meet the mechanical property requirements of blades. Therefore, it is urgent to find new alternative fibers with excellent mechanical properties and high corrosion resistance [14]. The elastic modulus of BF is 10–25% higher than that of GF. It has the advantages of having a low weight, a high strength, and corrosion and fatigue resistance [15]. Moreover, the energy consumption of BF is about 3–4 kW·h/kg [16], which is lower than 6–8 kW·h/kg of GF [17]. Therefore, BF is a perfect substitute for GF in the clean energy sector and has a high application value [18].

There are still several technical and equipment issues in the BF and its composites manufacturing business, including poor product performance stability, backward production equipment, and insufficient standardization of the manufacturing process. This paper discusses the composition and production process of BF, summarizes the properties, interface problems, and modification methods of BF, and creatively introduces the application of BFRP in the clean energy sector and power grids in order to accelerate the further development of BF and its composites in production and application.

## 2. Chemical Composition and Manufacturing Technology of BF

Since BF is made of pure natural ore by direct melt drawing, the chemical composition of basalt ore and the production process will have a large impact on the physical and chemical properties of BF.

### 2.1. Chemical Composition of BF

**Figure 1:**
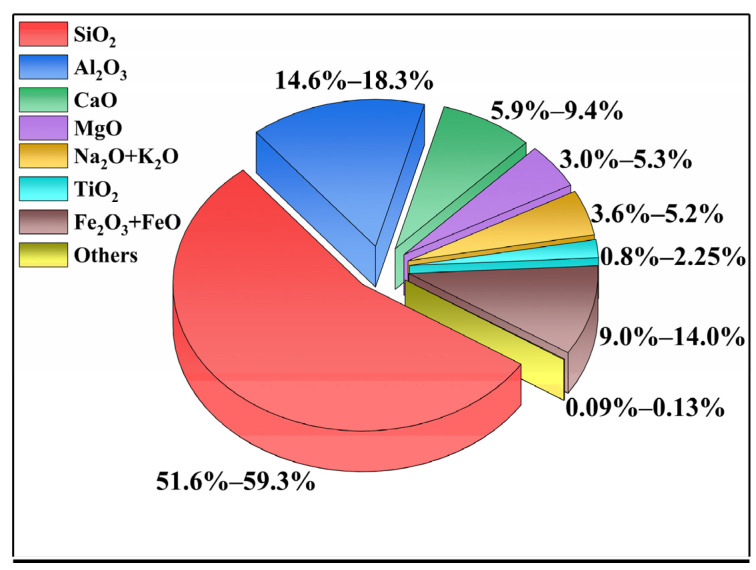
Chemical composition of basalt fiber (BF).

BF is rich in silicon, aluminum, iron, magnesium, calcium, sodium, and potassium oxides. Figure 1 shows the content of each component of BF. The content of SiO_2_ in the BF is approximately 51–59%, which provides BF with good chemical stability and mechanical properties. The content of Al_2_O_3_ is 14–18%, which provides BF with good thermal stability and durability. The content of Fe_2_O_3_ + FeO is generally 9–14%, which makes the fiber body brown and improves the high-temperature resistance of the fiber. Compared with GF, BF contains special components, such as MgO, Na_2_O, K_2_O, and TiO_2_, which is the reason that BF has better water and corrosion resistance [19,20]. Moreover, Kuzmin et al., found that the addition of Li_2_O or Na_2_O would reduce the fiber-forming temperature and expand the fiber-forming temperature range [21]. Basalt is distributed in many regions of the world, such as the Ural Mountains in Russia, Indonesia, and the western United States, and there are abundant reserves in almost every province in China, such as Heilongjiang, Sichuan, Shandong, Fujian, Anhui, Shanxi, Ningxia, and Hebei. Therefore, the specific chemical composition of BF will vary according to the geographical location of the basalt stone.

Due to the composition of natural basalt ore, BF faces a series of problems, such as a large dispersion of product properties and poor stability. Wu and his team at Southeast University proposed the basic concept of multivariate homogeneous blending to improve the homogeneity and stability of BF [22]. The research showed that when the SiO_2_ content was 50–57%, the tensile strength and homogeneity of BF were optimized. In this case, the chemical and mineral compositions had the greatest impact on the tensile strength. When the content of SiO_2_ was greater than 57% or less than 50%, the tensile strength and homogeneity of BF was not good. In this case, the tensile strength of the BF was mainly determined by the melting properties or the formability of the fiber, respectively. In addition, SiO_2_ and Al_2_O_3_ in the BF led to a compact structure of the basalt glass network and improved the tensile strength of the BF, while the alkali metal and alkaline earth metal oxides K_2_O, Na_2_O, MgO, CaO, and TiO_2_ led to a loose glass network structure and adversely affected the tensile strength. Therefore, understanding the influence mechanism of the chemical composition of BF on its properties is the key to carrying out the selection and improvement of BF.

### 2.2. Manufacturing Technology of BF

**Figure 2:**
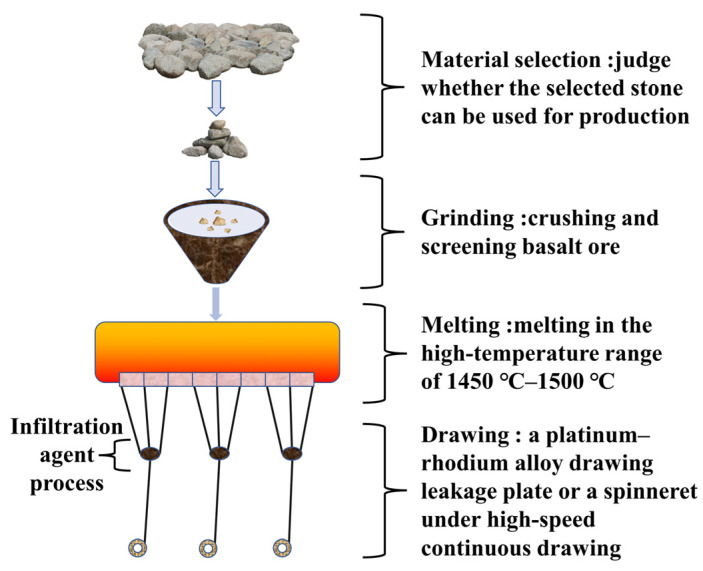
BF production process [23,24].

The production of BF generally requires four basic steps, which are as follows: material selection, grinding, melting, and wire drawing, as shown in Figure 2.

The production process of BF has been continuously optimized, and the output quality has been increased. The development of the production process has mainly experienced two stages. The crucible method, also known as the two-step method, is the main method used in the first stage. The first step of the method is to melt a certain proportion of raw materials at a high temperature to prepare a spherical drawing material. The second step is to remelt the spherical material in a crucible, homogenize it, and draw it into the final product. The method is cumbersome, time-consuming, energy-consuming, and complex in the production plant, but the homogenization effect is better and less likely to cause clogging of the leakage plate. The second stage is mainly the tank kiln method, also known as the one-step method, which completes melting and wire drawing together. This method is similar to the manufacturing process of GF, with the advantage of a lower energy consumption, no additives, and a lower cost than GF or CF [25]. Compared with the two-step method, the one-step tank kiln method eliminates the ball making process and has the advantages of a simple process, lower energy consumption, less pollution, less land occupation, and a high production rate, but the homogenization effect is poor and the leakage plate is easily blocked. However, the tank kiln method can avoid the problems of incomplete homogenization by controlling the proportion of raw material components, and appropriate raw material components can reduce the melt viscosity, prevent recrystallization, and avoid the problem of leakage blockage. In the process of tank furnace melting, it is worth noting that the basalt melt’s heat permeability is just 0.031–0.038 W·m^−1^·K^−^^1^ [26]. Generally, electric melting is used to improve the melting efficiency of basalt raw materials. However, the high content of iron oxides in the raw materials leads to more significant erosion of the heating electrode. In addition, the electric melting heating method will also accelerate the enrichment of iron oxides at the kiln bottom and accelerate the erosion of the platinum–rhodium alloy leakage plate. On 29 March 2019, China’s first 8000 t/a continuous BF tank kiln production line with completely independent intellectual property rights was successfully put into operation in Deyang City, Sichuan Province, which marked a breakthrough in the development of BF tank kilns in China. At the same time, this also shows that the development of large-scale tank kilns is an important trend of BF production in the future.

Based on the tank kiln method and the different drawing and forming processes of fibers from the melt, there are some other production methods, such as the flame injection method and the centrifugal injection method. With the support of these different production methods, BFs with diameters ranging from tens of nanometers to tens of microns can be mass produced industrially. In 2019, China’s BF output reached 14,200 tons, the demand was approximately 7500 tons, and the domestic market scale exceeded 100 million yuan [27]. Although the industrial production of BF has been basically realized in China, the bottlenecks of the raw materials, kilns, molding, and other production technologies involved in the stabilization, large-scale production, and high-performance of BF are still the main factors hindering the development of the continuous BF industry [22]. It is believed that with the development of BF production technology, a BF production process with a lower energy consumption and more stable performances will be developed.

## 3. Surface Morphology and Properties of BF

### 3.1. Surface Morphology Characteristics of BF

**Figure 3:**
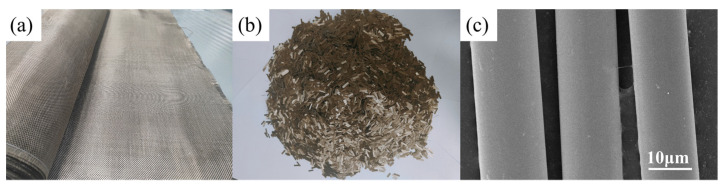
Shape and appearance of BF, (**a**) BF sheet, (**b**) short-cut BF, and (**c**) scanning electron microscopy image of BF.

BF is brown, has a smooth cylindrical appearance, and has completely rounded interfaces. This is because basalt is a single raw material, and almost no volatiles are produced in the process of melting and molding, which makes the basalt melt basically uniform. In the process of drawing and cooling the molten basalt into solid fibers, it will be affected by surface tension and shrink into a circle with the smallest surface area [22]. In terms of size and thickness, Parmar found that by changing the drawing speed of the fiber and the temperature of the melt, a wide range of fibers can be produced. Finer products will be obtained at high temperatures and speeds, and coarser products will be obtained at low temperatures and speeds [28]. The relevant shape and appearance of BFs are shown in Figure 3. In terms of the microstructure, a BF is a network skeleton with SiO_4_ tetrahedra as the main chain, in which Al is present as interstitial atoms, and other metallic elements are connected to the surrounding regions of the Si-O tetrahedra main chain as cations. Metal ions on the surfaces of the BFs associate with protons or hydroxyl groups from air and water due to the failure to meet the coordination number, resulting in the hydroxylation of the surface.

**Figure 4:**
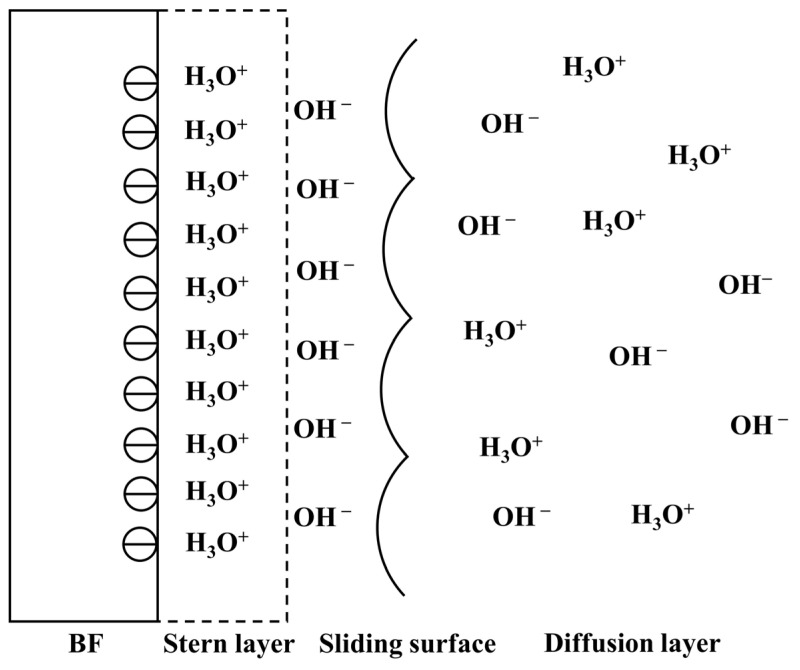
Schematic diagram of bare BF electric double layer [29].

By measuring the zeta potential of bare BF, researchers have found that the surface of bare BF is weakly acidic, and the zeta potential is negative. As with silicate fibers, the zeta potential of BF decreases with the increase in pH. When Al^3+^ forms an aluminum–oxygen tetrahedron with non-bridging oxygen in its network, an excess negative charge will be generated. To make the BF surface electrically neutral, positive ions will be attracted from the surrounding environment, and these positive ions will, in turn, be diffused at the two-phase interface to form an electric double layer [29]. The schematic diagram of the electric double layer is shown in Figure 4. According to the zeta potential analysis, the surface of bare BF is negatively charged, and the cationic surfactant can be directionally and efficiently adsorbed on the surface of a single fiber through charge action. Therefore, cationic surfactants have become the most commonly used lubricant agents in BF sizing [29]. Because bare BF will absorb low-energy impurities from the environment, the surface energy of bare BF is lower than the theoretical surface energy of silicate glass but still higher than the surface energy of the resin matrix. In addition, the polar component of the surface energy of bare BF is above 90%, while the commonly used resin matrix is dominated by the dispersion component, and the polarity difference between the two is large, which in turn leads to its poor bonding ability [30]. To maximize the intermolecular forces between the BF and the matrix resin, the surface energy of the BF should be as high as possible and have a polar component similar to that of the matrix resin. The surface modification of BF will be discussed in Section 4.2.

### 3.2. Properties of BF

#### 3.2.1. Mechanical Properties

The mechanical properties of fibers include the tensile strength, elastic modulus, elongation at break, and other parameters. Table 1 shows that the tensile strength and elastic modulus of BF are significantly higher than that of E-GF, which have little differences compared with those of S-GF. The elongation at break of continuous BF is slightly larger than those of CF and aramid fiber, which is lower than that of S-GF [31].

#### 3.2.2. Thermal Properties

As shown in Table 2, the softening point and thermal expansion coefficient of BF are higher than those of E-GF. The maximum service temperatures of GF and CF can only reach 380 °C and 500 °C, respectively, while BF can withstand both temperatures above 700 °C and below −260 °C [33]. This rare property allows BF to be used in special applications, such as aerospace and fire insulation [33]. Ying et al. studied the tensile strength of BF and GF at 300 °C, 400 °C, 500 °C and 600 °C, respectively. The results show that the tensile strength of BF is higher than that of GF at different temperatures, and the longer the time at high temperature, the more obvious the advantage [34].

**Figure 5:**
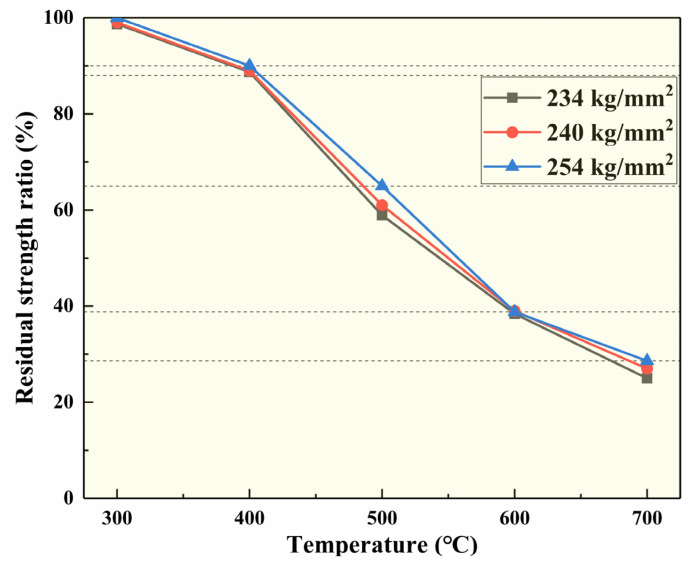
Residual strength ratio of BF fabric at different temperatures [32].

In addition, researchers have studied the residual strength ratio of different fabric types of BF at different temperatures [28]. The results are shown in Figure 5. The BF could still maintain an 88% to 90% residual strength ratio when the continuous working temperature reached 400 °C. At 500 °C, 600 °C, and 700 °C, the residual strength fraction could reach 65.0%, 38.8%, and 28.6%, respectively, which fully illustrates the excellent heat resistance of BF [32].

#### 3.2.3. Corrosion Resistance

Basalt is a type of silicate ore, so BF will have natural compatibility with silicate. Alkaline elements in basalt, such as magnesium, titanium, sodium, and potassium, provide it with excellent alkali corrosion resistance. It is highly suitable for the manufacture of composite materials with special requirements for corrosion resistance, such as wind turbine blades [35,36].

**Figure 6:**
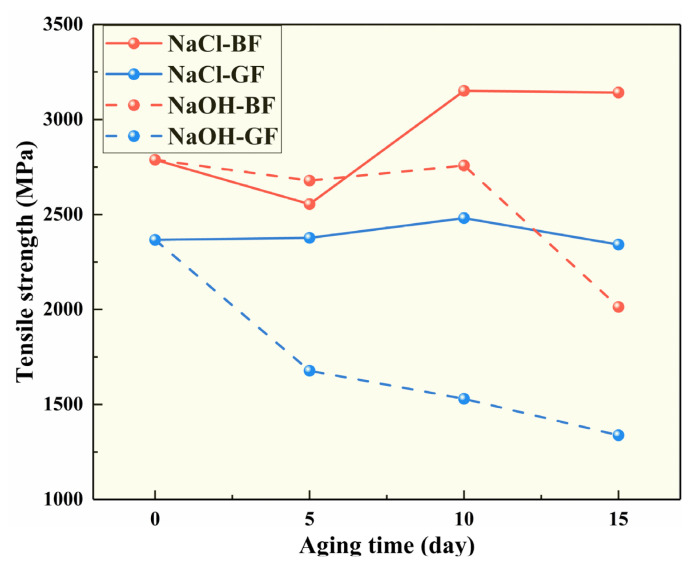
Salt alkali aging test results of different fibers.

The authors’ team investigated the salt and alkali corrosion resistances of BF and GF by subjecting BFs and GFs with diameters of 15 μm to aging tests in NaCl and NaOH solutions for 15 days and measuring their tensile strengths every 5 days. As shown in Figure 6, the tensile strength of BF was higher than that of GF in the whole aging process. In the NaOH solution, the tensile strength of the two fibers decreased to varying degrees, but the decline in the BF was less than that of the GF, indicating that the alkali corrosion resistance of BF was better than that of GF.

Researchers have improved the chemical corrosion resistance of the fiber by changing the proportion of basaltic raw materials, developing salt- and alkali-resistant sizing agents, and constructing metal oxide coatings. Lipatov et al. changed the proportions of the structure by adding ZrO_2_, ZrSiO_4_, and other components to the basalt raw stone, which enhanced the salt and alkali resistance of the BF to a certain degree [37]. Scheffler et al., showed that the cross-linked coating structure could more effectively improve the tensile strength and alkali corrosion resistance of the fibers [38]. Rybin et al., added zirconia coatings to the BF surfaces by a sol–gel method, and the results showed that the deposition of two zirconia protective coatings increased the salt and alkali corrosion resistances of the BF [39].

#### 3.2.4. Dielectric Properties

As shown in Table 3, BF has good dielectric properties. The volume resistivity of BF is 1 × 10^12^ Ω·m, which is an order of magnitude higher than that of GF. The range of dielectric constants from 100 Hz to 1 MHz is approximately 0.092–0.007; thus, it can be widely used in the production of printed circuit boards in the electronics industry. BF contains less than 20% conductive oxides by mass. After special sizing treatment, its dielectric loss (tanδ) is 50% lower than that of GF, making it ideal for use as an insulation material in electrical equipment [22]. BF has higher electrical insulation and permeability to electromagnetic waves than GF, providing it with very promising application potential in high-voltage electrical insulation materials, low-voltage electrical devices, antenna fairings, and radar radio devices [32].

#### 3.2.5. Environmentally Friendly Production

BF raw materials are derived from natural basalt ore, which is environmentally friendly. There is no need to add new components in the production process of BF, and there are no emissions of hazardous substances, such as boron and other alkali metal oxides. Moreover, its composition is the same as that of basalt ore, so there is no pollution to the environment after degradation [41]. Furthermore, because BF is resistant to oxidation, it can be employed as a catalyst carrier in wastewater treatment and gas purification. By improving the contact chance between the catalyst and pollutants, the catalytic performance may be fully exploited, and there is no secondary pollution [42]. We should continue to work on BF applications in environmental protection, such as environmental–friendly building material and automobile exhaust gas treatment, in the future.

## 4. Characteristics and Improvement Methods of BF Interface

Currently, China attaches high importance to the research of the development of BF and its composite materials, and relevant research institutions have been increasing their investment in the research and development of the high-tech BF industry. However, compared with developed countries such as Russia and the United States, China’s BF industry still has considerable room for development, and there is still much work to be done in terms of technology development and product application. Therefore, the research on the surface modification technology of BF and the bond strength of the reinforced interface is of great significance for the widespread promotion and application of BFRP.

### 4.1. Characteristics of BF Interface

In BFRP, the fiber and matrix play the role of load bearing and load transfer, respectively, and the interface is the small area between them, which is usually only a few microns or even a few nanometers, acting as a bridge. Good interface bonding is conducive to the transfer and dispersion of an external load between the fiber and the matrix, preventing stress concentration, and effectively blocking and inhibiting the further expansion of cracks [43]. In contrast, when the interface bonding is poor, the external load cannot be transmitted well in the composite, resulting in stress concentration at the defect, which destroys the mechanical properties of the composite [44]. Therefore, the properties of composites are closely related to the bonding degree of the interface [45,46].

In terms of electrical performance, when the interface between the fiber and matrix is poor, interface defects will occur. Under the action of high electric intensity, partial discharge will induce the growth of electric or water branches [47,48,49]. Meanwhile, the improvement of the interface bonding degree can inhibit the polarization of the resin long chain and reduce the polarization loss, and thus reduce the leakage current. In addition, the closer bond between the fiber and the resin matrix can also make the electric field of the composite interface layer more uniform and improve the breakdown strength [50].

However, the surface of BF is smooth and inert, resulting in poor adhesion between the BF and resin matrix and a low interfacial bonding strength, which has a significant impact on the properties of the materials, especially the electrical insulation properties. These shortcomings significantly limit the realization of the advantages of BF, which in turn affects the further development and application of BF and its composites. Therefore, the research on the surface modification technology of BF and the enhancement of its interfacial bond strength is of great significance for the widespread promotion and application of BFRP.

### 4.2. Surface Modification of BF

Surface modification of BF mainly has two types, physical and chemical. Physical modification refers to changing the surface roughness, specific surface area, and other microstructural characteristics of BF, increasing the mechanical meshing center to enhance the physical bonding between the BF and the resin matrix. Chemical modification of BF is a process of introducing chemical functional groups onto BF, improving the surface activity of BF. The chemical reaction between BF and the matrix resin can enhance the chemical bonding between the two phases to enhance the bonding strength of the interface. Currently, surface modification technology of BF mainly includes acid–base etching, surface coating, plasma treatment, coupling agent treatment, nanomaterial treatment, and sizing agent treatment.

#### 4.2.1. Acid–Base Etching

In the acid–base etching method, the BF surface is etched with an acid or base to form grooves or protrusions, which changes the smoothness and roughness of the fiber surface. Wei et al. [51] treated BF with NaOH to strengthen resin matrix composites. Through scanning electron microscopy, it was observed that the resin polymer adhered to the fiber surface, indicating that the bonding degree between the fiber and resin was improved. Mechanical properties, such as the tensile and impact strengths of the composites, were found to be improved to different degrees by mechanical tests.

To study the differences in the etching results of acids and bases on BF, Xie et al. [52] treated BF with 1 mol/L HCl and NaOH. The results showed that the specific surface area of the nonetched BF was 1.1526 m^2^/g, while those that etched the BF etched with acid and base increased by 1.0674 and 3.7849 m^2^/g, respectively, indicating that the surface roughness of fiber had significantly improved, and the effect of alkali etching was superior.

Although acid–base etching can improve the bonding degree between BF and resin, it also causes damage to BF. Wang et al., used NaOH to treat the BF mesh. The tensile breaking forces of the etched BF mesh reduced from 1.55 kN to 0.96 kN in the warp direction, and the strength retention ratio in the weft direction decreased by 26.9% [53]. Nasir et al. studied the etching effect of H_2_SO_4_ on BF. They found that the mechanical characteristics of BF declined as the etching duration increased [54]. The acid–base etching modification can improve interfacial adhesion at the expense of BF performance.

#### 4.2.2. Surfactants Coating

A fiber surface coating forms a bridge between the fiber and the resin, allowing stress transfer in the interface layer and directly affecting the mechanical and electrical properties of the composites. The method of BF surface coating modification is relatively simple and has strong designability. It mainly improves the fiber surface state by constructing an excellent coating on the BF surface. There were many materials that can be used as active agents, and Xu et al. [55] used Tween-80, sodium dodecyl sulfate, and cetyltrimethylammonium chloride (CTAC) to make three types of active agents, all with 1.5% mass fractions, to modify the surface coating of BF, and the contact angle of the modified BF decreased from 133.57° to 74.24°, 67.48°, and 62.52°, respectively. The hydrophilic properties were improved to varying degrees. Film-holding experiments showed that the CTAC-modified BF had the highest film-holding rate, film-holding volume, and film-holding strength. Zhu et al. [56,57] from Xi’an Jiaotong University prepared composites with la-ethylenediaminetetraacetic acid (EDTA)-coated treated p-BFs and bisphenol A dicyanate (BADCy) for mechanical testing. The results showed that the initial decomposition temperature of the modified composite was 145 °C higher than that before treatment. Moreover, when the mass fraction of the La-BF was appropriate, the bending modulus could reach more than 4.19 GPa.

In addition, the chemical corrosion resistance of the BF surface coating structure is the key to giving full play to the chemical corrosion resistance of BF. However, the research on special activators for the salt alkali corrosion resistance of BF coating structures is relatively scarce, which hinders the popularization and application of BF to a large extent. Therefore, it is necessary to combine the surface characteristics and application requirements of BF to build a BF surface coating with a good bonding strength and chemical corrosion resistance.

#### 4.2.3. Plasma Treatment

Plasma modification technology can make the fiber surface rough without damaging the fiber itself, under appropriate treatment conditions. Li et al. [58] considered factors such as the pressure, discharge time, and discharge power, treated BF with low-temperature plasma, and found that the fiber surface roughness increased, the content of C(1s) decreased relatively, the content of Si(2P) increased significantly, and the capillary effect increased.

In addition, plasma can improve the surface properties of fibers by introducing active groups on the surfaces of fibers through chemical reactions. Wang et al. [59] treated the surface of BF with mixed N_2_ and H_2_ gas. The results not only showed that the surface of the modified fiber was rougher but also that active groups, such as -OH and -NH_2_, were introduced onto the fiber surfaces. Sun [60] treated BF with air low-temperature plasma at a constant pressure and discharge time with discharge powers of 50, 100, 150, 200, and 250 W. It was found that with the increase in the discharge power, the dynamic and static friction coefficients increased, the contact angle decreased from 82.3° to 38.5°, and the fiber surface wettability was improved.

#### 4.2.4. Coupling Agent Treatment

Coupling agent treatment is a commonly used fiber modification method when it is difficult to achieve direct chemical bonding reactions between fibers and a matrix. Generally, the coupling agent contains two different types of functional groups, which can react with the fibers and the matrix to form an indirect chemical bond between them and improve the bonding strength. For example, after modifying BF with the silane coupling agent aminopropyltriethoxy silane, it was found that the number of Si–O bonds on the fiber surface significantly increased compared with that before modification [61]. In addition, Arslan et al. treated BFs with three different silane coupling agents and then studied the mechanical properties of the treated fiber-reinforced-poly(butylene terephthalate) composites. The results showed that epoxy silane coupling agents could increase the tensile and flexural strengths of the composites by 30.25% and 11.65%, respectively, and the reinforcement effect was the most significant. This showed that the formation of a covalent bond between the fiber and the matrix improved the properties of the composites [62].

Liu et al. [63] prepared BF/poly (lactic acid) composites by the vacuum perfusion method. The aminopropyltriethoxy silane coupling agent successfully linked BF and poly (lactic acid), improving the interfacial bonding strength of BF/poly (lactic acid) composites. Yu et al. [64] used amino-silane coupling agents to modify BF and then applied them to BF-reinforced polyamide 6,6 composites. The interfacial shear strength (IFSS) of BF/PA6,6 composites modified by mono-amino, diamino, triamino, and long-chain amino-silane coupling agents increased by 16.17%, 27.92%, 44.67% and 76.78%, respectively, as compared to unmodified samples. This indicated that as the number of amino groups increased, IFSS improved steadily, and the modification effect of long-chain amino-silane coupling agent was the best.

#### 4.2.5. BF Coated with Nanoparticles

Nanoparticles have large surface activity and are nano-sized particles. They can be attached to the surfaces of BFs through physical or chemical action to improve their adhesion to the matrix. Liu [65] treated BF with SiO_2_ nanoparticles and found that the specific surface area of the fiber surface increased significantly, and the friction coefficient also increased. Nanoparticles can also produce an anchor effect between fibers and resin, which can effectively increase the bonding forces between the fibers and the matrix, resulting in composites with superior mechanical properties. For example, Li et al. [66] found that the impact strength and interlaminar shear strength of BF composites with added nano-SiO_2_ increased by 139.73% and 27.25%, respectively.

In addition, Xu [67] of Zhongbei University also studied the nano-modification of BF. Nano-silica, carbon nanotubes, and graphene were grafted onto the surfaces of BFs mainly by infiltration of a slurry and dopamine, which improved the BF interfacial adhesion and wear resistance to a certain extent. When reduced graphene oxide/Ni particles are sprayed on the fiber surface, the BF will obtain certain microwave absorption properties. Kim et al. prepared functional composites by grafting carbon nanotubes onto the surfaces of BFs to improve their conductivity and explored their application prospects in electric heating and magnetic shielding [68].

The above studies showed that SiO_2_ and other nanoparticles have great application space for improving the surface properties of BF.

#### 4.2.6. Infiltration Agent Treatment

An infiltration agent is a mixture of film-forming agent, lubricant, antistatic agent, and coupling agent. The coating of an infiltration agent is a very important step in the industrial production process of BF. After coating, the water in the infiltration agent is removed from the BF by a yarn-drying process, so that the infiltration agent has a series of physical and chemical effects to improve the mechanical properties of BF. Moreover, the formula of the infiltration agent is called a “black box” by the BF industry. The infiltration agent is not only the core technology of industrial production but also an important factor restricting the development of domestic BF.

The influence of each component of the infiltration agent on the mechanical properties of BF is different. The orthogonal test results of Xing et al. [69] showed that the main film-forming agent had the greatest influence on the tensile strength, followed by the auxiliary film-forming agent, and the silane coupling agent had the least influence. Moreover, when the contents of the main film-forming agent and auxiliary film-forming agent were 6% and 1%, respectively, and the content of silane coupling agent was 0.8%, the effect was the best. Compared with the traditional infiltration agent, the infiltration agent modified with special materials tended to be more effective. He et al. [70] modified the wetting agent with nano-SiO_2_, -ZrO_2_, and -CeO_2_ particles and found that the composite prepared by modified infiltration with ZrO_2_ particles had the best high-temperature stability and residual interlaminar shear strength. The composites prepared using CeO_2_-particle-modified infiltration agents exhibited better high-temperature stability at the later stage of high-temperature experiments. The composites prepared using the SiO_2_-particle-modified infiltration agent had the worst high-temperature stability and the lowest residual interlaminar shear strength.

Compared with GF, BF has a large surface energy and strong polarity, while the polarities of commonly used resin matrices are weak, and there is a significant difference between them. Therefore, to improve the bonding strength between fibers and a matrix, the surface energy and polar component of the infiltration agent are particularly important. This is the intermediate bridge between the BF body, which is dominated by polar components, and the resin matrix, which is dominated by dispersion components. Chen et al. [29] prepared a bisphenol-F epoxy Pickering emulsion to treat BFs by surface wettability and flocculation modification of gas-phase SiO_2_, with the cationic surfactant CTAC and nonionic surfactant F108. In the subsequent experiments, it was found that the water absorption ability and flexural strengths of the composites were improved, as well as the hygrothermal aging properties.

Currently, there is relatively little research on the special wetting agents for BF, and most of the treatment methods in industrial production are borrowed from traditional GF treatment. Due to the significant differences between the surface characteristics of BF and GF, the applicability of the traditional infiltration treatment method is weak, and the excellent mechanical properties of BF cannot be brought into full play. Targeted improvements must be made based on the unique surface characteristics and specific application scenarios of BF. Good interfacial bonding properties will further exploit the excellent performances of BF, which in turn will enhance the overall performance of BFRP.

## 5. Applications of Basalt Fiber-Reinforced Polymer

**Figure 7:**
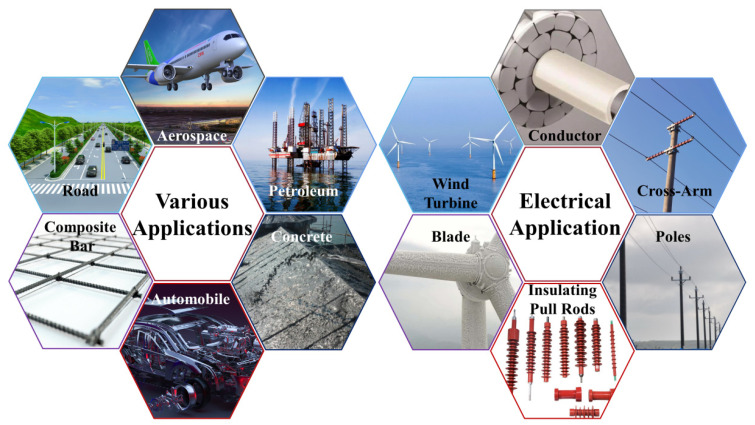
Application fields of BF.

Composites with BF as a reinforcement have better chemical stability than GF composites and lower costs than CF composites. Therefore, as shown in Figure 7, BFRP have wide application prospects in the modern industry and are colloquially known as new materials in the 21st century.

### 5.1. Applications of BFRP in the Electrical Sector

#### 5.1.1. New Conductors

For high-voltage distribution lines with voltage levels of 220 kV and below, the main restriction on the transmission capacity of the line is the thermal stability limit. The maximum allowable operating temperature of aluminum conductor steel-reinforced cables (ACSR) is 70 °C, which takes into account the recrystallization annealing effect of ACSR and the sag of conductor thermal expansion. Moreover, as a wire core, metal not only increases the hysteresis loss but is also prone to electrochemical corrosion with aluminum. Therefore, with the development of fiber-reinforced composites, it is necessary to find high-performance composite substitutes for traditional wire cores.

Among the composite transmission wires, the composite wire with CF as the core was developed earlier, and the technology has become more mature. Due to the low weight, high strength, and high-temperature resistance of CF, the use of CF instead of steel to make wire cores in the power industry is conducive to reducing the sag and wire loss. However, the price of CF conductors per unit length is 2–3 times that of steel-cored aluminum strands with the same specifications, which has significantly hindered the large-scale popularization and application of CF conductors [71]. Compared with CF, BF has a high-cost performance and strong corrosion and fatigue resistance, so it has significant application advantages in wire core materials. In addition, BF can operate in the temperature range of −260 °C to 700 °C. Therefore, BF composite cores can overcome the influence of bad weather, such as line icing, on the conductor performance.

The main function of a wire core made of BF or CF composite is to bear varied stresses on the conductor and increase its strength. The aluminum-stranded wire at the periphery is mainly employed to transfer electric energy. However, both CF and BF have significant defects when used alone as wire core materials. For example, the cost of CF is high, the texture of BF is brittle, and the tensile strength and elastic modulus of BF are lower than that of CF. BF and CF mixed as the core material can not only reduce the cost but can also ensure the performance, which can become a direction for the development of new conductors in the future. Compared with ordinary conductors, composite core conductors have the following advantages: (1) high strengths. The tensile strength of ordinary steel wire is 1240 MPa, the tensile strength of high-strength steel wire is 1410 MPa, and the tensile strength of a BF and CF mixed mandrel of a composite core conductor can exceed 2200 MPa. (2) Low weights. The density of the composite core material is approximately 1/4 of that of steel. Based on the span of 500 m, the vertical load of a tower can be reduced by approximately 750 kg to conserve tower materials and reduce the investment. (3) Low losses. Since there is no eddy current magnetic loss of the steel core, the loss of the transmission line itself is reduced, so the operation cost is reduced [72].

#### 5.1.2. Wind Turbine Blades

The installed wind power generation capacity has reached new heights in recent years, and the single-unit capacity has gradually developed from the kilowatt level to the megawatt level. With the increase in the single-unit capacity, the wind turbine blade has gradually entered the 100-m era. Traditional wind turbine blades are mainly composed of GF-reinforced composites, but when the length of a wind turbine blade exceeds 40 m, the GF-reinforced materials cannot meet the blade stiffness, shear resistance, and weight requirements. In addition, for an offshore wind turbine, the wind turbine blade operates in an extremely harsh marine environment for a long time, which requires the blade material to have a high strength, fatigue resistance, and salt spray corrosion resistance. To meet the application requirements in complex environments, large wind turbine blades must be made from CF-reinforced structures, which are extremely expensive. There is an urgent need to find new alternative fibers with excellent mechanical properties and high corrosion resistance [14].

BF has excellent mechanical properties, high-temperature resistance, and acid and alkali corrosion resistance, and it can operate in harsh environments, such as the sea, for long periods. Moreover, BFRP has the advantages of a low cost, high designability, and easy processing, which can meet the requirements of wind turbine blades [34,35]. Therefore, using BF or BF/CF to make wind turbine blades can greatly reduce the cost and improve the performance.

#### 5.1.3. Insulating Pull Rods

An insulating pull rod is one of the important components of an SF_6_ insulated circuit breaker, which mainly plays the role of supporting, transmitting forces, controlling line on–off switching, and insulating. Due to the frequent opening and closing times of circuit breakers, the insulating pull rod is required to have not only high insulation and mechanical properties but also strong fatigue resistance [73]. Insulating pull rods were first formed by curing epoxy-resin-impregnated GF, but these had the disadvantages of high weights, brittleness, and lack of wear resistance. In addition, aramid fiber composites are also used in the production of insulated pull rods, but they have the disadvantages of high cost, complex material forming technology, and high requirements for processing technology. Thus, they need to be optimized.

BF plain cloth treated with a coupling agent can replace the traditional GF cloth. BF is mixed evenly with epoxy resin, an accelerator, a curing agent, a toughening agent, and other auxiliary materials, and then wet winding can be used to prepare the insulating pull rod of electrical equipment, which can be used in high-voltage circuit breakers. The insulating pull rod made of BF composite exhibited no flashover, breakdown, or other phenomena after withstanding a voltage test for 5 min at 80 kV, which effectively solved the problems of the short-circuit discharge caused by defects of insulating pull rods caused by traditional GF-reinforced materials. Thus, the BF composite could significantly improve the reliability of insulating pull rods [74].

#### 5.1.4. Composite Cross-Arms

Composite cross-arms use composite materials to replace the original steel cross-arms, making full use of the advantages of composite materials, such as their good insulation performances, high strengths, low weights, excellent corrosion resistances, and convenient installation. This is another innovative application of new composite materials in transmission lines beyond composite insulators. However, the traditional GF-reinforced epoxy-resin-based composite cross-arms have some shortcomings, such as low elastic moduli, brittleness and ease of cracking, weak long-term temperature resistance, weak water resistance, and rapid aging in humid and hot environments, which greatly limit the application and promotion of composite cross-arms in some areas of China. BF has a higher volume resistivity, tensile strength, elastic modulus, and working temperature than conventional E-GF. At the same time, it also has many excellent properties, such as a high insulation strength, acid and alkali corrosion resistance, high-temperature resistance, and low water absorption. It is highly suitable for manufacturing composite cross-arms with special requirements for the mechanical properties and weather resistance.

#### 5.1.5. Composite Poles

Electric poles are among the important infrastructures in a power system, and the reliability of their structural performances is of great importance for the safe operation of the power grid. Based on the materials, the traditional distribution network poles can be divided into wooden poles, cement poles, and metal poles. These traditional poles have many disadvantages. Wooden poles easily rot under water exposure for a long period, are vulnerable to insects and birds, and consume wood resources. The manufacturing process of cement poles is complex, the weights are large, and the power consumption for manufacturing steel bars is large. Although metal poles have firm structures and good conductivities, they are large in size. Due to the excellent mechanical and chemical properties composite materials have, composite poles can be considered to replace traditional poles. Composite poles can be divided into composite bar reinforced poles and full composite poles. Of these, composite bar reinforced poles are widely used because of their low cost and mature technology. In the 1990s, countries around the world began to use GF-reinforced reinforcements to replace traditional reinforcements and make poles with cement matrices. The GF composite pole has the advantages of a low manufacturing cost, high tensile strength, and excellent chemical corrosion resistance. However, due to the poor anti-fatigue performances of GF itself, a GF composite pole will have problems such as a strength decline and severe aging under harsh environments at high temperatures and humidity for long periods, which is not conducive to the safe operation of a power grid.

Related research has shown that BFRP bars have higher adhesion and bond strengths to concrete than GFRP bars exposed to acid, alkali, and salt environments for 30, 60, and 90 days [75]. When the researchers added 0.5%, 1.5%, and 2% BF to polymer concrete, the tensile strength could be increased by 2%, 32%, and 35%, respectively [76]. Similarly, when BF with contents of 0.1%, 0.5%, 1%, and 1.5% was added into concrete mixtures as a reinforcement, the flexural strength was increased by 8%, 34%, 39%, and 75%, respectively [77]. These results showed that BFRP-bar-reinforced composite poles can operate stably in humid environments for long periods, and they have outstanding corrosion resistance and mechanical strengths. They are especially suitable for coastal regions, northwestern China, and other high saline areas [78].

### 5.2. Applications of BFRP in Other Sectors

#### 5.2.1. Petrochemical Industry

BF is mostly used in petroleum casing and pipes in the petrochemical industry. BF pipes have a density of around 1/4 that of regular metal pipes, which allows to save staff and material resources in production, shipping, and installation [79]. In terms of performance, the excellent bending strength, bending modulus and elongation at break of BF enable BF casing and tubing to easily bypass obstacles without using steering joints, and to smoothly pass through directional wells with tiny radius of curvature. In terms of service life, BF casing is not needed for thermal insulation and anti-corrosion treatments due to its strong thermal and chemical stability, which can reduce the maintenance cost, and prolong the service life of the equipment. The average service life can reach 60~80 years [80].

#### 5.2.2. Construction Engineering

Along with its firm structure, superior wear resistance, and low water absorption, BF, similar to basalt ore, has a unique advantage in the building business. BF offers a high strength-to-cost ratio when compared to other fibers [81,82]. Furthermore, because basalt ore and building materials, such as cement and fly ash, have comparable element compositions, BF and cement-based composites have a higher degree of commonality, making them more suitable for use in construction [83,84].

Traditional cement-based composites are brittle and short-lived. Using a suitable proportion of BF, the strength and toughness of cement-based composites can be enhanced, and the formation and extension of cracks in composites can be decreased [85].

#### 5.2.3. Automobile Industry

Energy-saving and environmental protection are the most considered requirements of the automotive industry at present, and the green and cheap characteristics of BF fully satisfy these demands. The lightweight and high-strength BF composites have an advantage over the iron car body and car shell. Furthermore, its superior water resistance and friction resistance have a wide range of applications in the automobile industry. Farzin et al. [86] studied the impact resistance of BF and CF composites. The findings of the low-speed impact tests demonstrate that BF composites have better impact resistance. Huang et al. [87] compared the friction and impact properties of BF and GF composites and found that the friction and impact properties of BF composites were 33.8% and 7.45% higher, respectively, than GF composites.

## 6. Conclusions and Prospects

Based on the composition and production processes of BF, this paper summarizes the properties, interface problems, and modification methods of BF and introduces the applications of BF in clean energy sector and power grids. The main conclusions and prospects are as follows:

(1) BF exhibits good comprehensive performances and environmental friendliness. It is one of the important materials to replace traditional GF and CF. Determining the influence mechanism of the chemical composition, mineral composition, and melting properties of BF on the fiber strength is helpful for optimizing the production process of BF and improving its properties. However, the previous research on BF was mainly focused on applications, and the mechanism of its stabilization and high strength was not examined.
(2) The interfaces of fiber-reinforced polymers are the key to ensuring the full performances of two-phase materials and improving the comprehensive properties of the materials. The interaction mechanisms of interfaces are highly complex, and they often act together and are affected by a variety of forces. Therefore, the interface bonding phenomenon should be explained with a variety of theories. There are many methods of BF modification, which can improve the bonding properties of composite interfaces to a certain extent.
(3) BF is a basic material, from which many industrial materials can be derived. This can not only drive the development of relevant emerging industries but can also provide new material support for the upgrading of relevant fields of the Chinese economy. With the rapid development power industry, BF, which has high insulation properties, high strength, corrosion resistance, high-temperature resistance, and fatigue resistance, as a new promising green material, will have great potential in power grid construction. It is necessary to vigorously conduct application research of BFRP and promote the industrialization process.
(4) In the future, it is necessary to focus on stabilization strategies and formation mechanisms of the excellent properties of BF, in combination with a variety of key factors in the production of BF. Currently, the widespread application of BFRP requires excellent interfacial bonding properties. Therefore, an in-depth discussion on the pretreatment and modification methods of BF is still the focus of future research. At the same time, the research methods of interface properties in materials and physics can be used to provide a reference for the interface research of FPR composites. In addition, in order to promote the further development of BFPR, demonstration projects can be established in petrochemical, construction, aerospace, automobile, power, and other industries to accumulate practical engineering experience.

## Figures and Tables

**Table 1:** Comparison of mechanical properties between BF and other fibers [26,32].

Fiber Type	Basal Diameter (μm)	Density (g·cm^−3^)	Tensile Strength (MPa)	Modulus of Elasticity (GPa)	Elongation at Break (%)	Price (USD·kg^−1^)
Basalt fiber	6–21	2.65–3.00	3000–4840	79.3–93.1	3.1	2.5–3.5
E-glass fiber	6–21	2.55–2.62	3100–3800	72.5–75.5	4.7	0.75–1.2
S-glass fiber	6–21	2.46–2.49	4590–4830	88–91	5.6	5–7
Carbon fiber	5–15	1.78	3500–6000	230–600	1.5–2.0	30
Aramid fiber	5–15	1.44	2900–3400	70–140	2.8–3.6	25

**Table 2:** Comparison of thermal properties between BF and other fibers [26,32].

Fiber Type	Maximum Working Temperature (°C)	Softening Point (°C)	Thermal Conductivity (W·m^−1^·K^−1^)	Thermal Expansion Coefficient (10^−6^·°C^−1^)	Heat Loss Rate (%)
Basalt fiber	700	960	0.031–0.038	8.00	1.91
E-glass fiber	380	850	0.034–0.040	5.40	0.32
S-glass fiber	300	1056	0.034–0.040	29.00	-
Carbon fiber	500	-	5–185	0.05	-

**Table 3:** Comparison of dielectric properties between BF and other fibers [32,40].

Fiber Type	Volume Resistivity (Ω·m)	Frequency of Dielectric Loss Tangent (MPa)	Relative Permittivity
Basalt fiber	1 × 10^12^	0.0050	2.2
E-glass fiber	1 × 10^11^	0.0047	2.3
Carbon fiber	2 × 10^−5^	-	-
Aramid fiber	3 × 10^13^	-	3.85

## Data Availability

The original data needed to reproduce these discoveries cannot be shared, because these data are also part of the ongoing research. The original data used to support the results of this study can be obtained from the communication author.
